# An Alternative Theoretical Approach to Escape Decision-Making: The Role of Visual Cues

**DOI:** 10.1371/journal.pone.0032522

**Published:** 2012-03-12

**Authors:** Veronika Javůrková, Arnošt Leoš Šizling, Jakub Kreisinger, Tomáš Albrecht

**Affiliations:** 1 Department of Zoology, Biodiversity Research Group, Charles University, Prague, Czech Republic; 2 Center for Theoretical Study, Charles University and Academy of Sciences of the Czech Republic, Prague, Czech Republic; 3 Institute of Vertebrate Biology, Academy of Sciences of the Czech Republic, Brno, Czech Republic; University of Sussex, United Kingdom

## Abstract

Escape enables prey to avoid an approaching predator. The escape decision-making process has traditionally been interpreted using theoretical models that consider ultimate explanations based on the cost/benefit paradigm. Ultimate approaches, however, suffer from inseparable extra-assumptions due to an inability to accurately parameterize the model's variables and their interactive relationships. In this study, we propose a mathematical model that uses intensity of predator-mediated visual stimuli as a basic cue for the escape response. We consider looming stimuli (i.e. expanding retinal image of the moving predator) as a cue to flight initiation distance (FID; distance at which escape begins) of incubating Mallards (*Anas platyrhynchos*). We then examine the relationship between FID, vegetation cover and directness of predator trajectory, and fit the resultant model to experimental data. As predicted by the model, vegetation concealment and directness of predator trajectory interact, with FID decreasing with increased concealment during a direct approach toward prey, but not during a tangential approach. Thus, we show that a simple proximate expectation, which involves only visual processing of a moving predator, may explain interactive effects of environmental and predator-induced variables on an escape response. We assume that our proximate approach, which offers a plausible and parsimonious explanation for variation in FID, may serve as an evolutionary background for traditional, ultimate explanations and should be incorporated into interpretation of escape behavior.

## Introduction

Accurate timing of escape, as determined by flight initiation distance (FID; distance between prey and predator when escape begins), enables prey to avoid a lethal encounter with an approaching predator. In accordance with the theoretical optimality model [1], [2] and its extended versions [3]–[5], prey adjust FID based on a cost/benefit ratio in order to achieve maximal fitness. Numerous studies [6] have demonstrated reduced FID in situations where risk of predation is low and/or cost of escape high. In these cases, measures of FID provided relatively strong arguments supporting the optimality paradigm. It is extremely difficult to obtain precise fitness consequence estimates of decision-making (i.e. optimality model parameters) in nature, however, and most empirical studies do not evaluate the sufficiency of empirical data gained for optimality models adequately [7]. As a consequence, there is currently no well-established complementary interpretation framework available to the dominant view of FID in terms of economic rationality derived from normative models.

Decision-making is inherently a function of cognitive, physiological and neurobiological processes at the proximate level [8]–[10]. However, heuristics (or rule-of-thumb logic) used by prey during the decision-making process do not always correspond with the economic rationality assumed by most optimality models [11]–[13]. These aspects are predominantly considered as a “black box” in evolutionary based studies on decision-making [14]. Nevertheless, incorporating a proximate insight into the decision-making process theoretical model may prove a fruitful strategy, providing parallel (i.e. not necessarily mutually exclusive) frameworks for interpretation of several phenomena and stimulating theoretical as well as empirical progress in this field [12], [15]–[17].

Behavioral decision making and adopted anti-predator behavior depends highly on the acquisition of acoustic or visual signals from the environment [8], [18], [19]. Quality of visual perception in particular has been identified as a predictor of inter-specific variability in anti-predator performance, including vigilance [20], [21], predator detection [22] and, most recently, escape response [23]. To date, there have been only a few studies that have explored escape behavior incorporating proximate explanations and that consider escape responses triggered by visual stimuli [24]–[28]. In these studies, escape behavior was considered as elicited by looming stimuli (i.e. projection of the angle subtended by an approaching predator's frontal profile onto the retinal image) and escape response as generated by a threshold size and/or speed of the “looming image” on the retina [26], [29], [30]. Moreover, firing level intensity of specific visual neurons was observed to correlate with looming expansion [9], [31] and physiological activity of muscles related to escape behavior [32]–[34]. In other words, visual stimuli activate the escape response and, therefore, could provide a suitable keystone for a proximate interpretation of the escape decision-making process.

The ultimate approach has traditionally been used to explain changes in FID in relation to changing vegetation cover or directness of a predator's approach trajectory [6]. Vegetation cover and directness of trajectory should also affect both visual acuity and the retinal image of an approaching predator, i.e. they are likely to affect the looming stimuli. Indeed, many studies have documented a shorter FID for individuals in habitats surrounded by dense vegetation [35]–[37]. The effect of vegetation cover on FID is usually considered to be associated with a decrease in perceived risk due to a prey's inconspicuousness [38]; however, vegetation cover may also prevent accurate processing of visual information from the environment [39]–[41]. This obvious proximate explanation is generally underestimated in the context of FID.

**Figure 1:**
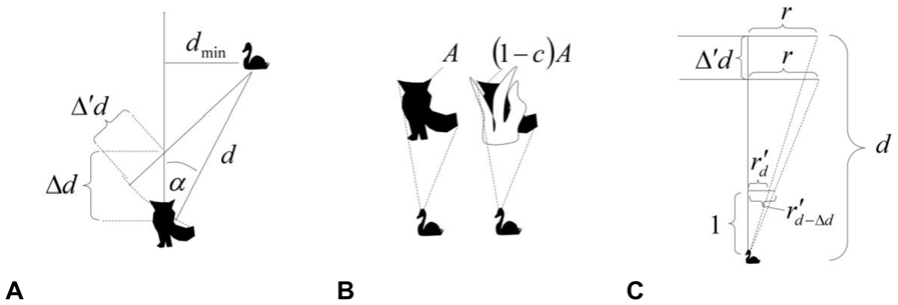
Relationships between predator frontal profile size, predator-prey distance and what the prey can actually see. (A) If a predator, at a predator-prey distance 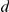, moves along a trajectory that bypasses the prey at distance 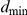, then each step of 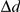 shifts the predator of 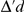 toward the prey. (B) The prey does not see the whole predator frontal profile size (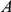) but only a portion 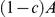, where 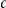 corresponds to actual vegetation concealment that obstructs the prey's view. The predator's frontal profile size (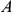) is calculated as a product of a profile shape specific coefficient (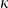) and the square of an effective diameter (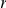), i.e.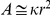. (C) The prey can apparently see the predator as if it was placed on a screen at a distance of one (see ‘1’ in the Figure). Consequently, the apparent size of the predator's frontal profile corresponds to apparent diameters 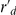 and 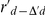, for the distances 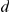 (before a step) and 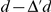 (after the step), respectively.

Similarly, the effect of directness of predator trajectory on FID may also be interpreted in two ways. Broom and Ruxton [5] have suggested that prey should either flee immediately a predator is detected, or stay motionless and rely on crypsis. The “motionless” strategy is more advantageous when the predator's trajectory bypasses the prey's position, since it intuitively decreases the probability of being detected by the predator. Based on the ultimate explanation, prey perceives a predator approaching tangentially as less of a risk. The proximate view, however, proposes that visual processing of an object (i.e. a predator) moving directly towards the prey causes a stronger cue for flight initiation. There is a lack of transversal shift in the retinal image in the case of a direct approach [42] and, therefore, we can conclude that expansion of retinal image is the more relevant cue, see [10], however, for limitations to visual processing of a directly moving predator in fiddler crabs (*Uca vomeris*). In contrast, the retinal image of a predator moving strictly tangentially (i.e. the predator does not approach the prey at all; see Figure 1) does not expand [42] and, therefore, the retinal image of a tangentially moving predator magnifies less than the retinal image of a predator moving directly toward the prey. This would suggest that the visual cue for escape response is weaker during a tangential approach and that the prey would, in consequence, delay its flight initiation [10], [26], [42]. Based on these proximate predictions, vegetation cover, which is supposed to constrain quality of visual acquisition, may have a stronger effect on escape decision-making in the case of a directly, rather than tangentially, approaching predator.

This study proposes a mathematical model based on simple visual processing of an approaching predator. Based on the model's predictions, we examine escape decision-making in incubating mallards approached by a human. The data revealed a negative correlation between FID and nest vegetation concealment when prey were approached directly. During a tangential approach, however, we observed no effect of vegetation concealment on FID. We believe that a description of the proximate mechanism behind a particular behavior is crucial for an understanding of observed variability in response to different risk factors. We suggest that our model is able to bridge the gap in our knowledge of proximate mechanisms of prey escape decision-making and that its incorporation into the ultimate framework could improve the interpretation of prey escape behavior and make it more biologically relevant.

## Methods

### Model and Theory

We assume that a prey's decision to escape is triggered by changes in the geometry of a visual signal. Such a signal is given by the apparent size of a predator's profile, *A*, which will increase with decreasing distance between prey and predator, *d*, and decrease with a decrease in actual size of the predator, 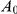 (i.e. 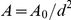; see Figure 1). If vegetation cover is involved then the predator profile, as seen by the prey, is reduced by nest vegetation concealment *c*, expressed as the proportion of the predator that is obscured by vegetation (see below), thus 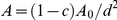. Intuitively, even a 100% increase in apparent predator profile would appear negligible if the predator is apparently small (this would also include a very distant predator). On the other hand, even a small proportional increase (e.g. 10%) in a large apparent predator profile will be noticeable to prey (this would also include a small predator at a short distance). Because ducks, like most birds, have very poor stereoscopic vision, we assume that an increase in apparent predator size 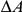 between two instants is the cue for a duck to take flight. 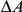 will be largest when a predator is heading directly toward the nest (direct approach; 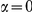; Figure 1) and will be zero if the predator passes the nest strictly tangentially (strict tangential approach; 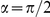; Figure 1).

Vegetation concealment *c*, predator directness 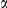, its actual size 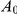, and distance *d*, all define a particular situation that uniquely determines the relationship between the cue to fly 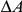, and any shift by the predator between two instants in which a duck analyses its surrounding 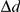 (Figure 1). This relationship obeys
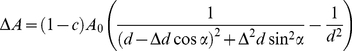
(1)(for details see Text S1). As the species-specific value for the cue to fly is met at the species-specific FID, we can modify eq. 1 by replacing *d* with FID, thus
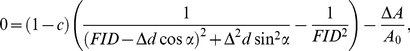
(2)which is numerically solved with respect to FID using the bisection method (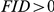) (see [43], and http://en.wikipedia.org/wiki/Bisection method). Symbolically, we can write the solution of the bisection method as a function of *c*, with three parameters 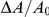, 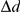 and 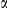. The notation then follows
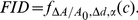
(2)The FID derived from eq. 2 is supposed to apply to all individuals of the focal species. However, in order to take individuality of prey into account, we include an individual specific term *I* into the FID (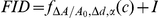). This allows for some individuals reacting before (and some after) reaching the critical value of FID. Since there is no reason for a zero mean value of individuality, the modeled FID obeys
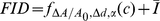
(3)where 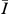 is a mean value of bias in the prey's individuality.

In the design scheme for the field experiment (see Figure 1 for details), the angle 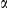 varied with type of predator approach toward the mallard's nest (i.e. tangential/direct). Variation depends on a minimum distance between the linear trajectory of the predator and the nest 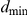, and follows
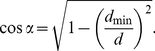
(4)If we predict that a change in apparent predator profile size (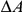) triggers an escape response (i.e. 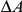 does not essentially vary among individuals) then the model suggests FID as a function of vegetation concealment 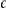, and four parameters 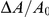, 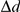, 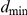 and 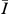. Thus
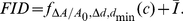
(5)In our experimental scheme, 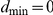 and 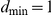 represent direct and tangential approaches, respectively.

The model was fitted to data based on the relationship between *c* and the difference between FID for direct and tangential approaches in order to reduce the number of free parameters to two (i.e. 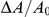 and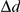). Thus, the model for the difference between direct and tangential FIDs obeys
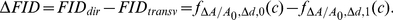
(6)This assumes (i) equal size of predator, (ii) equal predator speed during each experimental approach, and (iii) equal 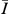 for direct and tangential approaches. If the predator varied in its size or speed then 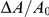 or 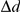 could not be treated as constant, and if 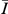 varied between direct and tangential approaches then 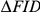 would be biased from the predicted value. Equation 6, therefore, was fitted by randomly varying 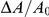 and 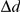 (between 0 and 1, and 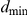 and 1, respectively). Four thousand pairs of parameters were randomly drawn and the sum of square residuals between them predicted (eq. 6; FID values extracted from eq. 2 by the bisection method) and observed values calculated. The 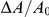 and 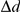 minimum sum of square residuals were taken as fitted if one thousand additionally drawn pairs of parameters did not provide a better fit (a smaller sum of square residuals). If one of the one thousand random pairs provided a better fit then a new set of a thousand pairs of parameters was drawn. The procedure ended when the last one thousand pairs of parameters did not provide a better fit. Proof that the minimum sum of square residuals lay within the range from which the parameters were taken was checked visually on the graphs. Afterwards, the parameters 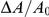 and 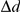 were used to compute particular relationships between *c* and FIDs for direct and tangential approaches. Mean individuality 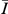 was extracted from the data on direct approach. For each c, the FID, without accounting for the mean individual reaction (i.e. 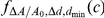 in eq. 5), was extracted from eq. 2 using the bisection method and 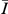 was computed as a mean across residuals between the predicted and observed FIDs. Finally, FIDs for tangential approach were computed by adding the value 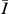 (eq. 5) to the FID extracted from eq. 2 (with 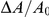 and 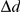 extracted from the previous fitting) using the bisection method. Three hierarchical fittings were thus performed: (i) fitting on data on the differences between direct and tangential FIDs with two free parameters; (ii) fitting on data on direct approach with one free parameter; and (iii) fitting on data on tangential approach with no free parameters. There is no mathematical reason why parameters extracted from the first fitting should suit the second or third fitting and why 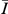 extracted from the second fitting should suit the third fitting as: (i) identical 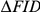
- concealment relationships (eq. 6) may originate from a variety of relationships between particular FIDs (e.g. a negative correlation between FID difference and vegetation concealment can result from decreasing or increasing of particular FIDs and decreasing FID-concealment relationships), and (ii) 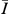 for tangential approach does not mathematically determine 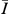 for direct approach. Hence, we test our model using three independent tests. First, we test for residuals of the fit of data on 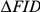
s; second, for residuals on the fit of data on the direct approach; and third, for residuals on the fit of data on the tangential approach. We would reject the model in the case that any residual showed a bias with vegetation concealment.

### Experiments

#### Ethics statement

The field experiment was carried out under permission no. 162 (15/2/2006), issued by the Ministry of Environment, on behalf of the Government of the Czech Republic.

#### Study area and model species

Field research was carried out from April to July 2006 and 2007 at four selected fishponds (area polygon covered 18 km^2^) situated in the Třeboň Biosphere Reserve (49°9′ N, 14°43′ E). We used mallards, a cryptically colored, ground-nesting, dabbling duck as a model species. Typical nesting habitat was represented by ten artificial fishpond islands (5–30 m wide, 100–300 m long) where all experimental nests were located. Vegetation on these islands consisted mainly of common reed (*Phragmites communis*), sedge grass (*Calamagrostis epigeos*), nettle (*Urtica dioica*) and bent-grass (*Carex* spp.).

#### Field procedures

Mallard nests were detected by walking slowly and systematically until incubating hens were disturbed, thus enabling us to localize the nests. We determined nest site vegetation characteristics for each nest by using a checkerboard-patterned (5×5 cm squares) plastic cube (20×20×20 cm) placed on top of the nest (see [38] for details). In order to obtain a value for nest vegetation concealment from the direction of the experimental predator's approach (see below), the percentage of squares covered with vegetation when viewed at 0.5 meters along the approach direction at a height corresponding to the female ducks head position (∼20 cm above ground) was scored (hereafter called “nest vegetation concealment”). We used a candler [44] to estimate the incubation stage for each clutch, enabling us to experimentally approach only nests with eggs at the same incubation stage (12–15 days) and to eliminate observed effect of current reproductive stage on FID [38], [45]. Nests with eggs at an advanced incubation stage were excluded from the experiment. In order to avoid the confounding effect of nest parasitism, we also excluded nests containing eggs of parasitic species (e.g. Tufted Duck (*Aythya fuligula*) and Gadwall (*Anas strepera*)). Moreover, we also excluded nests completely covered with vegetation (100% concealment) as there would be no looming stimuli to model in this case (see Model and Theory). All experimental nests (n = 17) represent a random sub-sample from four different study areas (see above).

#### Experimental design

Each nest was approached either directly or tangentially by the same observer (VJ) simulating a predator. All experiments were undertaken between 10:00 and 16:00 (CET). We recorded the FID for each approach (±10 cm), i.e. the distance of the predator (observer) from the nest at the moment when the female mallard started to flee. Direct approach was performed by slow (0.5 m/s) walking toward the nest. Due to the observed effect of bypass distance on FID [37], we standardized the tangential approach by setting a minimum perpendicular distance from the nest (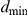 in Figure 1) equal to one meter and by walking slowly (0.5 m/s) along this trajectory. Predator sight was never targeted directly to the nest but above it at human eye level. To eliminate the confounding effect of head position on prey flight response [46], the observer's head was always oriented toward the movement trajectory during both experimental approaches (i.e. the observer's head did not turn toward the nest during a tangential approach). Individual types of experimental approach were applied in random sequence and the interval between experimental approaches at the same nest was not longer than four days, which enabled us to keep the incubation stage at the same phase during particular experimental approaches. In order to avoid the effect of starting distance (i.e. the distance between predator and prey when approach begins) on FID [47], we kept equal starting distances (ranging from 7 to 10 meters) for both experimental approaches to the same nest.

## Results

Mean FIDs ± SD were 2.4±1.04 m for a direct approach and 1.9±0.6 m (N = 17) for a tangential approach. The minimum approach distance (i.e. minimum FID) for both direct and tangential approach was 1 m, which corresponded to the minimum bypass distance used in our field experiment (i.e. 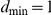). Maximum FIDs for direct and tangential approaches were 4 m and 3 m, respectively.

**Figure 2:**
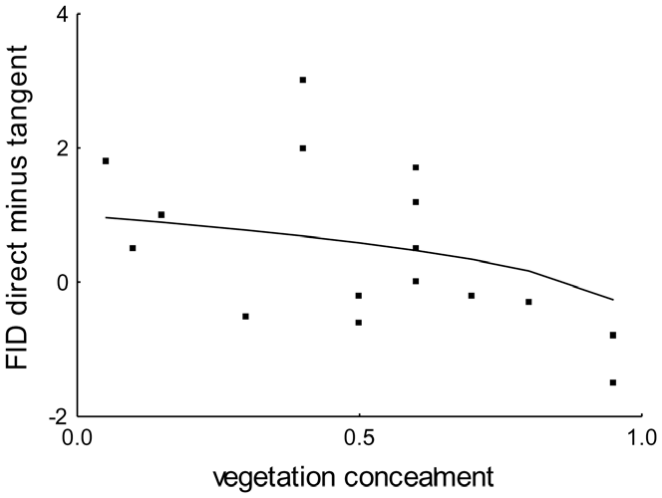
Observed (squares) and modeled (solid line) relationships between nest vegetation concealment and differences between two types of FID (direct minus tangential approach). N = 17, though some points overlap at symbols (see Data S1).

**Figure 3:**
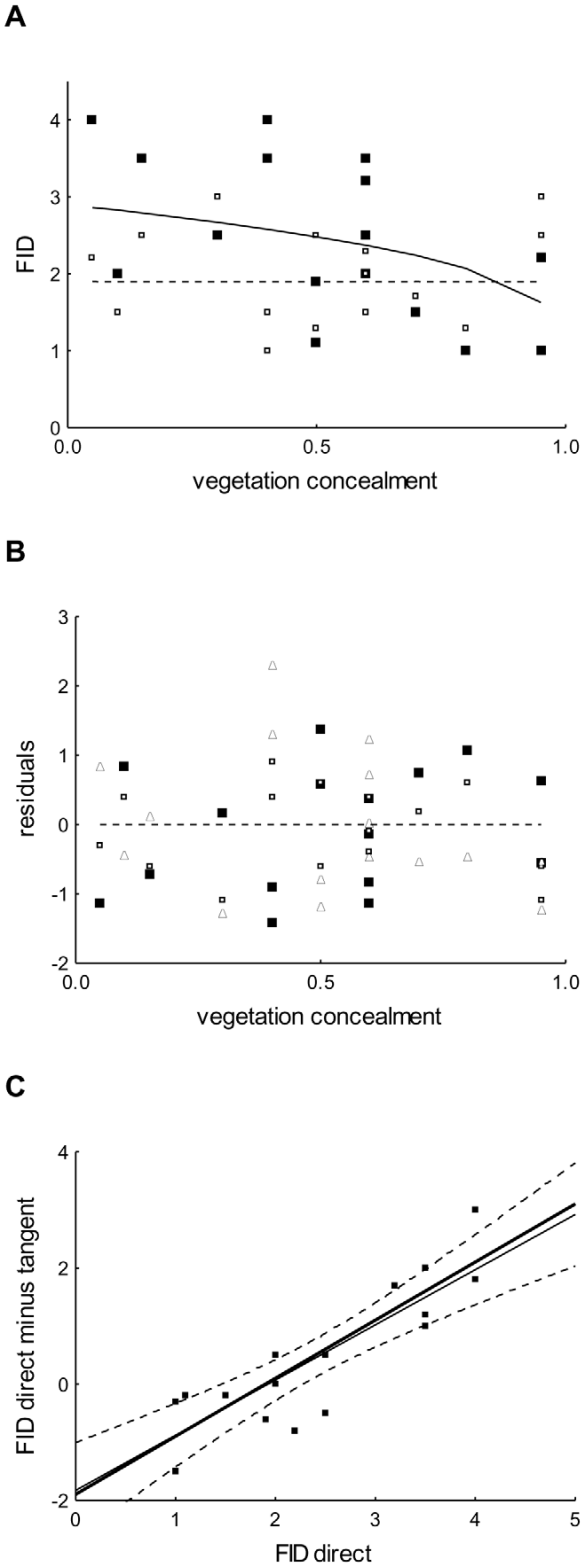
Particular relationships and their residuals. (A) FIDs/vegetation concealment relationships for tangential (observed  =  empty squares, modeled  =  dashed line) and direct (observed  =  solid squares, modeled  =  solid line) approaches. (B) Neither residuals between data and models for the tangential approach (empty squares) and the direct approach (solid squares) nor the direct minus the tangential approaches (triangles) show significant bias. (C) Observed (squares) and modeled (line) relationships between FID for a direct approach and the difference between FIDs for the two types of approach. N = 17 in all cases, though some points overlap at pictures symbols (see Data S1).

The modeled relationship (eq. 6) fitted to data on the difference between FIDs (fitted parameters: 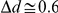 m, 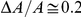) decreased with vegetation concealment (Figure 2). Models of particular relationships between vegetation concealment and FIDs for direct (fitted parameter: 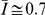 m) and tangential (no free parameter) approaches showed decreasing and constant curves (Figure 3A; phenomenologically, they could be approached with lines). None of the residuals between the fitted and observed FIDs showed significant bias (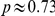, Wald. stat = 0.12, df = 1, N = 17; 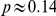, Wald. stat = 2.14, df = 1, N = 17; and 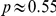, Wald. stat = 0.35, df = 1, N = 17 for direct, tangential, and the difference between both these approaches, respectively; GLM, 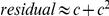, linear link) (see Figure 3B). Since there was also no evidence for any significant second order polynomials (i.e. 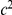) for particular approaches (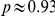, Wald. stat = 0.002, df = 1, N = 17; 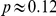, Wald. stat = 2.44, df = 1, N = 17; and 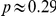, Wald. stat = 1.01, df = 1, N = 17, respectively), we did not reject the proposed model and considered it an appropriate proximate interpretation of escape decision-making.

For contrast, we attributed an artificially inverted vegetation concealment to each observation (e.g. the nest with 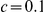 was given a new 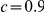; Figure S1), then ran the test for the deformed dataset. Both the residuals for fit on 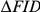 and FIDs for direct and tangential approaches were significantly biased (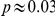, 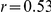,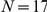;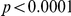, 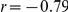, 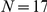 and 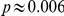, 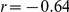, 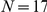, respectively) (see Figure S1). Thirty artificial data sets, where vegetation concealment was randomized across the observed nests, showed that all residuals on FIDs (for tangential approach) extracted from the randomized data were biased to a higher significance level than residuals extracted from observed data (i.e. *p* randomized was smaller than *p* observed). Of these, 16 residuals were significantly biased at a level of *p<*0.01 and five at *p<*0.05 (9 were non-significant). This model, therefore, is able to indicate a contrast between correct and deformed data.

Experimental data showed a significant relationships between (i) vegetation concealment and difference between FIDs (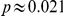, 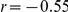, 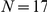) and (ii) between vegetation concealment and FID for direct approach (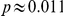, 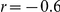, 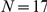), just as our model predicted. There were no significant relationships between vegetation concealment and FID for tangential approach (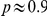, 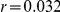, 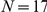). Furthermore, the plot of FID for direct approach against the difference between particular FIDs (Figure 3C) showed a linear relationship (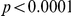, 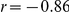, 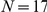; Figure 3C) with a slope of one (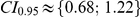). The FID for tangential approach, therefore, is independent of the FID for direct approach, as predicted.

## Discussion

Escape decision-making theory has long been interpreted mainly in terms of the ultimate fitness cost/benefit balance paradigm [1], [2]. Further, mathematical models based strictly on ultimate explanations for interpretation of the behavioral decision-making process are widely adopted and used by most behavioral ecologists [3], [4], [6]. This kind of approach has a certain degree of heuristic power; for example, observed inter- and intra-specific variability in escape response to identical risk factors is widely interpreted through their multiple (i.e. additive or interactive) effects [4], [48]–[50]. These ultimate variables, however, cannot be directly parameterized and are considered as hidden or latent variables which can only be correlated with observable behavior [7]. Such non-complex characteristics of ultimate approaches are taken into account in the most recent theoretical studies that incorporate a proximate insight into interpretation of decision-making processes during mate choice [51] or the decision to flee [52]. Accordingly, with respect to the above mentioned theoretical background, it is correct to assume that an understanding of the physiological mechanisms that trigger escape behavior are needed in order to produce proximate explanations that can eventually be applied in the sense of ultimate considerations [52], [53].

Several studies have confirmed that individuals primarily use information from their sensory systems [8], [19], [54] and that visually guided animals are able to precisely distinguish between a false and relevant visual signal in the environment [55], [56]. Surprisingly, even though empirical evidence exists for the escape response being closely linked with proximate cognitive and/or physiological mechanisms [9], [34], [57], this fact has mostly been ignored in theoretical models that evaluate escape decision-making [3]–[5]. To our knowledge, our study is the first that proposes a theoretical model predicting prey escape behavior based on looming stimuli and that includes both environmental- and predator-induced factors as model variables. By including the interactive effect of given factors affecting visual processing of an approaching predator, and through defined experimental conditions (e.g. no totally covered nests), we show that even a relatively complex escape response pattern where various risk factors interact [48]–[50] can be explained by a simple proximate mechanism.

Experimental data were consistent with the model's predictions regarding the interaction of effect of vegetation cover and directness of predator approach, i.e. FID increased with decreasing vegetation concealment during a direct approach but not during a tangential approach (see Figure 3A). These results can be interpreted with respect to the model's predictions in which we consider the different contribution of vegetation concealment to visual processing of a direct vs. tangential predator movement (eq. 5; Figure 1B). The fitted parameters (Data S1) indicate that the predator effectively covered a distance of 60 cms (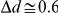) while a duck processed the visual information as indicating danger. As the speed of the “predator” was set at 0.5 m/s in our experiment, the duck's processing time is around 1 sec. This is in accordance with the mean individual reaction, which shifts average values of FID of around 70 cms toward longer distances (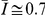), and with variability in individual reaction, which is about 1 m either side of the predicted value (Figure 3A). A value of 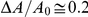 indicates that the predator's profile has to increase by approximately 20% with respect to actual predator profile, or in the case of a human being (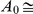
0.6 m^2^). This means that the trigger value (for the observed mallards) on the duck's “virtual screen” (see Figure 1 for details) at a distance of one meter is 0.12 m^2^ (or a two-dimensional angle of 0.12 steradians). For a small predator (e.g. 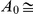
0.3 m^2^), therefore, the fitted parameter 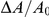 would be approximately 0.7 m^2^ (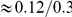), a conclusion that serves as a testable output of our theory (see also Software S1, Manual S1).

We assume that the trigger for escape behavior is the contrast in apparent predator size (equivalent to a two-dimensional angle in steradians) between two different instants (we assume the difference as species-specific). Such a trigger makes better biological sense than speed of the predator. If, for example, prey process a visual signal from two instants very close in time, the contrast in visual signal would be small and the prey probably would not notice any change. If, on the other hand, prey compare visual signals from two instants clearly separable in time, there is a higher chance of a noticeable contrast, which may then trigger a response. This resonates with the parental experience of a lack of progress in their children's development on a daily basis, whereas clearly visible changes are seen on photographs taken at Sunday picnics. Our model, therefore, not only takes account of predator speed but also the way in which prey separate visual signals from each other and how this affects visual stimuli. We assume that the values of these two factors result from predator/prey co-evolution.

Although previous studies have documented that FID decreases with increasing vegetation concealment, and have interpreted these findings in terms of the protective function of dense vegetation for the prey [36]–[38], [49], we suggest that the degree of vegetation cover limits the visual stimuli input [41], [58] and thereby affects FID. Moreover, our empirical data show a linear relationship between FIDs for a direct approach and the differences between particular FIDs (see Figure 3C). Our model, therefore, suggests that the visual appearance on a duck's retina of a predator moving directly toward prey will be different than the visual appearance of a tangentially approaching predator (but see [10], [26]).

Support for our proximate insight into escape decision-making is also provided by several studies demonstrating that variations in escape response are driven by certain constrains, such as the capacity of the visual system [23], [26], [28], [34] or the responsiveness of vision-related neurons [9], [34], [59]. Jabloński and Strausfeld [60] used a “looming image” projected on an insect's retina for modeling evolution of contrastive pattern in bird plumage coloration, a factor that appears to be crucial for foraging success in insectivorous flush-pursuing birds. This fact, in our opinion, indicates that proximate insight *per se* may have a predictive value for evolution and the interpretation of observed ultimate consequences.

In this study, we provide a simple proximate explanation for the effects of environmental- and predator-induced factors on FID. Although it is clear that escape decision-making is more complex than our model suggests [61], [62] (e.g. FID could also be affected by auditory stimuli in the case of dense vegetation concealment obstructing vision [63]–[65]), the good fit to data indicates that the overall pattern is well described by modeling visual stimuli and that escape is likely to be triggered by magnification of the predator's frontal profile. An ultimate evolutionary mechanism is thus likely to act through this proximate mechanism, making our model a useful tool for upcoming research of prey escape behavior.

## Supporting Information

Figure S1 **Results for artificially deformed data.** (A) Observed (squares) and modeled (solid line) relationships between nest vegetation concealment and differences between the two types of FIDs (direct minus tangential approach). (B) Particular FID/vegetation concealment relationships for tangential (observed  =  empty squares, modeled  =  dashed line) and direct (observed  =  solid squares, modeled  =  solid line) approaches. (C) All residuals between data and models for a tangential (empty squares) and direct approach (solid squares) and the direct minus tangential approaches (triangles) show significant bias. N = 17 in all cases, though some points overlap each other at symbols (see Data S1).
(TIF)

Text S1 **Derivation of** **eq 1** **.**
(DOC)

Data S1 **Dataset.**
(XLS)

Manual S1 **Software manual.**
(DOC)

Software S1 **Software.**
(EXE)
