# Supplementary figures and images for: An Alternative Theoretical Approach to Escape Decision-Making: The Role of Visual Cues
Source: PLoS One. 2012 Mar 12;7(3):e32522. doi: 10.1371/journal.pone.0032522 (PMC3299677; doi:10.1371/journal.pone.0032522)

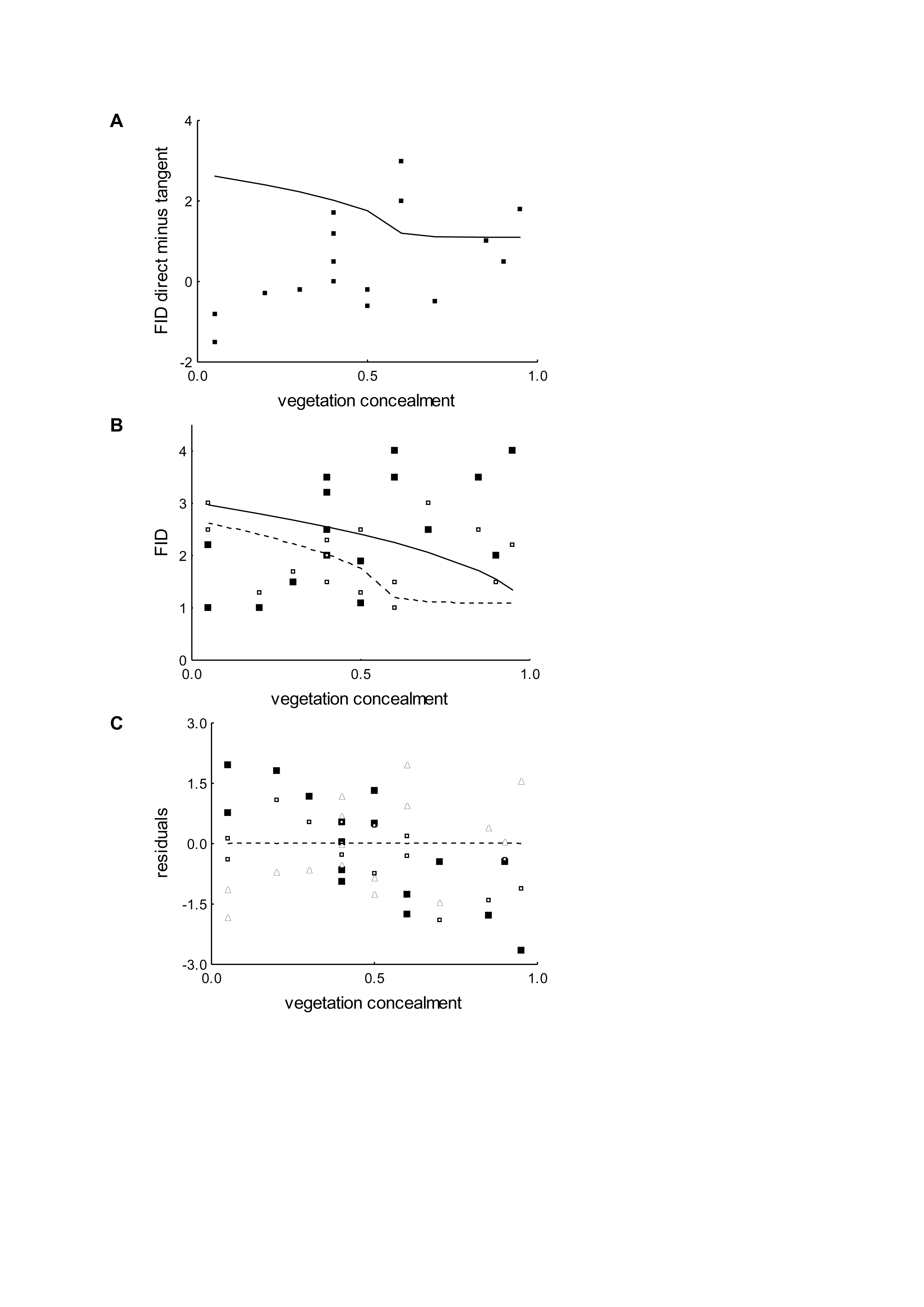

Supplement: Figure S1 — Results for artificially deformed data. (A) Observed (squares) and modeled (solid line) relationships between nest vegetation concealment and differences between the two types of FIDs (direct minus tangential approach). (B) Particular FID/vegetation concealment relationships for tangential (observed = empty squares, modeled = dashed line) and direct (observed = solid squares, modeled = solid line) approaches. (C) All residuals between data and models for a tangential (empty squares) and direct approach (solid squares) and the direct minus tangential approaches (triangles) show significant bias. N = 17 in all cases, though some points overlap each other at symbols (see Data S1). (TIF) [file pone.0032522.s001.tif]
