# Supplementary material for: An Alternative Theoretical Approach to Escape Decision-Making: The Role of Visual Cues
Source: PLoS One. 2012 Mar 12;7(3):e32522. doi: 10.1371/journal.pone.0032522 (PMC3299677; doi:10.1371/journal.pone.0032522)
Supplement: Text S1 — Derivation of eq 1 . (DOC) [file pone.0032522.s002.doc]

**Derivation of eq 1**

The size of the predator profile is given by the predator-profile specific coefficient $\kappa$ and the predator effective radius $r$ (see Figure 1). If the predator were circular, then $\kappa$ would equal $\pi$ and $r$ would be the circle radius. The predator radius apparently varies with the distance from the prey, $d$ (Figure 1). Geometrically, where $r'_{d}$ is a virtual radius of the predator. Hence, the apparent size of the predator's frontal profile follows , where $A_{0}$ is the actual size of the predator's frontal profile. Similarly for apparent profile of a predator after it approaches the prey of $\Delta'd$, . From the cosine formula, $\left(d-\Delta d\right)^{2}$ $\mathop{=}\limits d^{2}+\Delta^{2}d-2d\Delta d\cos\alpha$. It follows that $d^{2}+\Delta^{2}d-2d\Delta d\cos\alpha$ $=\left(d-\Delta d\cos\alpha\right)^{2}+\Delta^{2}d\left(1-\cos^{2}\alpha\right)$ $\mathop{=}\limits\left(d-\Delta d\cos\alpha\right)^{2}+\Delta^{2}d\sin^{2}\alpha$, for $\cos^{2}\alpha+\sin^{2}\alpha=1$. The apparent difference in the predator profile is thus which makes the eq. 1.
