# Supplementary material for: An Alternative Theoretical Approach to Escape Decision-Making: The Role of Visual Cues
Source: PLoS One. 2012 Mar 12;7(3):e32522. doi: 10.1371/journal.pone.0032522 (PMC3299677; doi:10.1371/journal.pone.0032522)
Supplement: Manual S1 — Software manual. (DOC) [file pone.0032522.s004.doc]

**Manual for Utility software**

1. Run the utility (click on “Software S1”)
2. Load data (click on ‘Load Data’ button; for data format see below, the file has two headlines (description and names of variables)
3. Set value of N (it is preset as 100). It is number of iterative steps. The higher N, the more accurate result. You can run this button repeatedly to increase accuracy of the fitting.
4. Save results
5. End the utility (click on ‘End’ button)

///////////////DATA SET///////////////////////////

Description of the dataset (Javůrková et al. 2012, doi: 10.1371/journal.pone.0032522)

Nest Concealment FID direct FID transv

30 1.5 1.7

85 3.5 2.5

40 2.5 2

40 3.2 1.5

5 2.2 3

60 3.5 1.5

70 2.5 3

60 4 1

5 1 2.5

20 1 1.3

5 4 2.2

40 3.5 2.3

40 2 2

50 1.9 2.5

90 2 1.5

50 1.1 1.3

20 1 1.3

//////////////////////////////End of the dataset
